# The Effect of Intradialytic Aerobic Exercise on Dialysis Parameters and Fatigue in Hemodialysis Patients: A Non-randomized Interventional Study

**DOI:** 10.7759/cureus.62498

**Published:** 2024-06-16

**Authors:** Yoga Lakshmi, Sasikala D, Santosh Varughese

**Affiliations:** 1 Medical-Surgical Nursing, Anurag University, Hyderabad, IND; 2 Medical-Surgical Nursing, The Tamil Nadu Dr MGR Medical University, Chennai, IND; 3 Nephrology, Christian Medical College, The Tamil Nadu Dr MGR Medical University, Vellore, IND

**Keywords:** fatigue, hemodialysis, non-randomized intervention, dialysis parameters, hemodialysis patients, intradialytic aerobic exercise

## Abstract

Introduction

Hemodialysis is the standard treatment for end-stage renal disease. However, patients receiving hemodialysis can become less active as a result of treatment, resulting in the accumulation of waste products. Intradialytic exercise improves the clearance of urea and creatinine by opening up vascular beds.

Materials and methods

We conducted a non-randomized interventional study to evaluate the effect of intradialytic aerobic exercise on dialysis parameters and fatigue among 295 hemodialysis patients selected through consecutive sampling (control group, n=147; experimental group, n=148) from two tertiary care centers. Baseline data on background variables and pre-test dialysis parameters (e.g., blood urea, creatinine, potassium, and hemoglobin levels) were assessed in both groups. Following connection to the hemodialysis machine, participants in the experimental group engaged in 15 minutes of intradialytic aerobic exercise per two hours of dialysis for a total of eight weeks, besides receiving routine care, compared to those in the control group. Post-test dialysis parameters were assessed for both groups at the end of the eighth week. The collected data were analyzed and tabulated using SPSS (IRB Inc., Armonk, New York).

Results

Intradialytic exercise led to significant improvements in post-test blood urea, creatinine, and fatigue in the experimental group (p<0.001). However, post-test serum potassium and hemoglobin levels remained relatively unchanged.

Conclusion

This study demonstrates the benefits of intra-dialytic aerobic exercise as a safe complementary therapy for a large population of dialysis patients, leading to better patient outcomes.

## Introduction

End-stage renal disease (ESRD) is a global health problem of growing concern, with approximately 1.2 million deaths reported in 2017 worldwide [1]. In India, the estimated number of deaths due to renal failure increased from 136,000 in 2015 [2] to 175,000 in 2018, with a prevalence rate of 129 per million population [3]. Hemodialysis (HD) is the foremost treatment modality for chronic kidney disease (CKD), accounting for 69% of all renal replacement therapies and 89% of all dialysis treatments [4]. Approximately >130,000 patients are reported to receive dialysis in India - a number that continues to rise by 232 per million population every year [5]. The rapid growth in the number of CKD patients receiving maintenance hemodialysis has led to exponentially improved survival rates. However, this increased level of hemodialysis is highly challenging for healthcare professionals providing comprehensive care while preventing potential related issues. Patients generally remain sedentary during hemodialysis, which is usually scheduled for three sessions per week, with each session lasting three to four hours.

Despite significant improvements in the life expectancy of CKD patients receiving hemodialysis, their quality of life remains unsatisfactory due to their sedentary behavior. Certain comprehensive interventions (e.g., yoga, meditation, relaxation therapy, and fitness regimens) have the potential to reduce fatigue and, thus, enhance quality of life. Studies have reported the benefits of intradialytic exercise, such as improvements in functional status, dialysis adequacy, and quality of life [6,7]. Intradialytic exercise leads to the opening of the blood vessels and increased capillary surface area, resulting in increased muscle blood flow and favoring the large influx of uremic toxins from the tissue to the vascular compartment, which is filtered through the dialyzer during hemodialysis [8]. Moreover, the incorporation of intradialytic aerobic exercise by hemodialysis patients is relatively easy, inexpensive, and requires only minimal preparation. Considering the benefits of intradialytic exercise, we carried out a study to evaluate its impact on dialysis parameters and fatigue in patients receiving hemodialysis.

## Materials and methods

**Figure 1:**
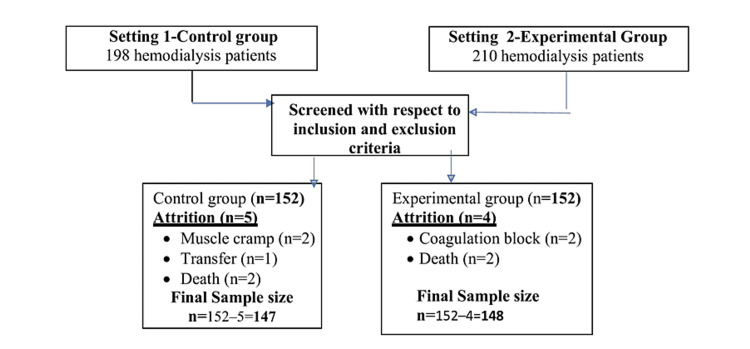
Schematic representation of sampling

A non-randomized interventional study was conducted at the dialysis facilities of two tertiary care centers from January 2021 to May 2022. Ethical clearance was obtained from the Institutional Ethics Committee of Apollo College of Nursing, Chennai. Sample size was calculated based on a study by Melanie et al. [9], with β = 80% (power), α = 0.05 (confidence interval), and urea mean post-intervention scores of 1.35 g/L ± 0.26 and 1.47 g/L ± 0.43 in the experimental and control groups, respectively. Thus, the predicted sample size was 138 for each group. To improve the generalization ability of the results and account for a potential attrition rate of 10%, 304 hemodialysis patients (152 for each group) were enrolled in the study. Patients were selected consecutively from both centers based on eligibility criteria. The study population included hemodialysis patients aged 20-59 years, undergoing hemodialysis for more than three months, scheduled for three times per week (three to four hours per session), and who were able to move their upper limbs. Hemodialysis patients who were unable to follow their prescribed exercise regimen or had blood coagulation on their dialysis filter during treatment, hemodynamic instability, experienced angina pectoris within the previous three months, a medical condition that precluded them from exercising, hemoglobin levels below 7 g/dL, serum potassium levels above 6 mmol/L, issues with arteriovenous fistulas, or active liver disease were excluded from the study. After attrition in both groups, 295 hemodialysis patients (148 in the experimental group and 147 in the control group) were finally included in the analysis, as shown in Figure 1.

The purpose of the study was explained to the patients, and informed consent was obtained from each study participant. All guidelines as per the declaration of Helsinki and good clinical practice were followed. The data regarding background variables were collected via interviews. Blood samples were drawn to test for blood urea, creatinine, potassium, and hemoglobin levels. The Fatigue Assessment Scale (FAS) developed by Michelsen et al. [10] was used to assess fatigue among patients. The FAS comprises 10 items (five items each for physical fatigue and mental fatigue) scored on a five-point Likert scale. The reliability of the tool was assessed and determined to be good (r=0.76). Following connection to the hemodialysis machine, the patients in the experimental group engaged in intradialytic exercise. The prescribed exercises included flexion (20 times/min) and extension (20 times/min) movements of the wrist, elbow, ankle, and clockwise (20 times/min) and anticlockwise (20 times/min) rotations of the ankle in addition to routine care. The exercises were demonstrated to the study participants in the experimental group by the researcher prior to initiating them. The exercises were done for 15 min in the first two hours of every session up to eight weeks. Patients in the control group received only routine care. Post-test blood urea, creatinine, potassium, hemoglobin, and fatigue were assessed at the end of eight weeks immediately after the final dialysis. Confidentiality was maintained throughout the experiment. SPSS version 22 (IBM Inc., Armonk, New York) was used to analyze the collected data. Descriptive statistics (e.g., frequency, percentage distribution, mean, and standard deviation) were used to describe the data. Inferential statistics included the Chi-squared test to assess the homogeneity of the study participants in both groups, the paired t-test to compare the dialysis parameters and fatigue levels between pre-test and post-test, the independent t-test to compare dialysis parameters and fatigue levels between the groups, and analysis of covariant (ANCOVA) to control the effect of hemoglobin on fatigue.

## Results

According to the distribution of background characteristics of the hemodialysis patients (Table 1), nearly half were aged 51-60 years, male, married, college graduates, had comorbidities of diabetes mellitus/ hypertension, family history of end-stage renal disease (ESRD, 65% and 62%), unknown etiology for chronic kidney disease (CKD, 25.17% and 25.68%), and were undergoing hemodialysis for more than three years (38.10% and 35.81%) in the control and experimental groups, respectively. There were no statistically significant differences observed with regard to selected background characteristics between the control and experimental groups.

**Table 1:** Frequency and percentage distribution of different background characteristics of hemodialysis patients in the control and experimental groups

Background characteristics	Control group (n=147)		Experimental group (n=148)		Chi-squared value	p-value
	f	%	f	%		
Age in years						
21-30	21	14.29	24	16.22	0.59	p=0.90
31-40	23	15.65	26	17.57		
41-50	41	27.89	37	25		
51-60	62	42.18	61	41.22		
Gender						
Male	77	52.38	82	55.41	0.27	p=0.60
Female	70	47.62	66	44.59		
Education						
Illiterate	10	6.8	10	6.76	0.2	p=0.98
Primary/middle	23	15.65	21	14.19		
High school/ higher secondary course	19	12.92	18	12.16		
Graduate and above	95	64.63	99	66.89		
Comorbidities						
Diabetes mellitus	24	16.33	22	14.86	0.41	p=0.94
Hypertension	15	10.2	14	9.46		
Both	80	54.42	86	58.11		
Others	28	19.05	26	17.57		
Family history of end-stage renal diseases						
Yes	96	65.31	92	62.16	0.32	p=0.57
No	51	34.69	56	37.8		
Risk factors of kidney disease						
Diabetes mellitus	24	16.33	22	14.86	0.84	p=0.93
Hypertension	39	26.53	35	23.65		
Polycystic kidney disease	15	10.2	19	12.84		
Unknown etiology	37	25.17	38	25.68		
Any other cause	32	21.77	34	22.97		
Duration of end-stage renal diseases						
<1 year	34	23.13	34	22.97	1.64	p=0.65
1-3 years	36	24.49	32	21.62		
4-5 years	20	13.6	28	18.92		
>5 years	57	38.78	54	36.49		
Duration of hemodialysis						
<6 months	14	9.52	11	7.43	0.92	p=0.93
6 months to 1 year	21	14.29	23	15.54		
>1-2 years	26	17.69	26	17.57		
>2-3 years	30	20.41	35	23.65		
>3 years	56	38.1	53	35.81		

Paired t-tests showed a significant reduction in blood urea and creatinine levels in both groups (p<0.001), as seen in Table 2, which could be attributed to the effect of hemodialysis. However, the mean difference between pre-test and post-test urea levels in the experimental group (38.06 mg/dL) was comparatively higher than that of the control group (8.96 mg/dL). Post-test fatigue was significantly lower in the experimental group than in the control group (p<0.001). Independent t-tests revealed a significant difference in post-test blood urea and fatigue scores between the two groups, as seen in Table 3. This difference could be attributed to the effect of the intradialytic exercise in enhancing urea clearance, thereby reducing the fatigue related to urea accumulation. No significant difference in serum potassium or hemoglobin was observed between the groups. The ANCOVA test in Table 4 revealed the estimated means of posttest fatigue after controlling the effect of hemoglobin on fatigue were 24.345 (control group) and 32.034 (experimental group). The experimental group exhibits a significant difference in fatigue mean compared to control group due to intradialytic exercise with an estimated mean difference of 7.68 (p<0.001), with 0.21% of variance (i.e adjusted R square = 0.207) even after controlling the effect of hemoglobin (0.03% variance at p=0.384). 

**Table 2:** Comparison of dialysis parameters before and after intradialytic exercise between the control and experimental groups SD - standard deviation; SE - standard error; p - calculated probability; Sr - serum

Variable	Group	Test	Mean	SD	Mean difference	SE	Paired 't'	p-value
Blood urea in mg/dL	Control	Pre-test	99.17	20.87	8.96	2.37	3.78	<0.001
		Post-test	90.21	22.25				
	Experimental	Pre-test	99.82	20.4	38.06	1.99	19.05	<0.001
		Post-test	61.76	12.18				
Sr creatinine in mg/dL	Control	Pre-test	6.82	1.26	0.99	0.17	5.95	<0.001
		Post-test	5.82	1.66				
	Experimental	Pre-test	6.37	1.44	0.68	0.18	3.70	<0.001
		Post-test	5.70	1.69				
Sr potassium in mEq/L	Control	Pre-test	4.63	1.32	0.12	0.13	0.91	0.37
		Post-test	4.75	0.99				
	Experimental	Pre-test	4.72	0.95	0.003	0.112	0.30	0.98
		Post-test	4.71	1.01				
Sr hemoglobin in g/dL	Control	Pre-test	8.15	1.49	0.13	0.19	0.66	0.50
		Post-test	8.02	1.92				
	Experimental	Pre-test	8.30	1.86	0.15	0.18	0.82	0.41
		Post-test	8.45	1.42				
Fatigue	Control	Pre-test	33.12	8.36	1.13	0.711	1.59	0.11
		Post-test	31.98	6.91				
	Experimental	Pre-test	33.96	7.46	9.57	0.62	15.42	<0.001
		Post-test	24.39	7.80				

**Table 3:** Comparison of dialysis parameters before and after intradialytic exercise between control and experimental groups. SD - standard deviation; SE - standard error; p - calculated probability; Sr - serum

Variable	Test	Group	Mean	SD	Mean Difference	SE	Independent 't'	p-value
Blood urea in mg/dL	Pre-test	Control	99.17	20.87	0.64	2.4	0.26	0.79
		Experimental	99.82	20.4				
	Post-test	Control	90.21	22.25	28.46	2.08	13.64	<0.001
		Experimental	61.76	12.18				
Sr creatinine in mg/dL	Pre-test	Control	6.82	1.26	0.43	0.16	2.71	0.007
		Experimental	6.37	1.44				
	Post-test	Control	5.82	1.66	0.11	0.19	0.56	0.58
		Experimental	5.7	1.69				
Sr potassium in mEq/L	Pre-test	Control	4.72	0.95	0.09	0.19	0.66	0.51
		Experimental	4.63	1.32				
	Post-test	Control	4.75	0.99	0.04	0.12	0.33	0.74
		Experimental	4.71	1.01				
Sr hemoglobin in g/dL	Pre-test	Control	8.15	1.49	0.14	0.19	0.72	0.47
		Experimental	8.3	1.86				
	Post-test	Control	8.02	1.92	0.43	0.19	2.17	0.031
		Experimental	8.45	1.42				
Fatigue	Pre-test	Control	33.12	8.36	0.84	0.92	0.91	0.36
		Experimental	33.96	7.46				
	Post-test	Control	31.98	6.91	7.59	0.86	8.85	<0.001
		Experimental	24.39	7.8				

**Table 4:** Effect of intradialytic aerobic exercise on fatigue in the experimental group while controlling for the effect of hemoglobin df - degree of freedom; F - the effect size; * Partial Eta squared  - how large of an effect the independent variable(s) had on the dependent variable a. R Squared = .213 (Adjusted R Squared = .207); a. Covariates appearing in the model are evaluated at the following values: posthb = 8.2414.

Source	Type III sum of squares	df	Mean square	F value	Sig.	Partial Eta squared *
Corrected model	4294.929^a^	2	2147.464	39.478	0	0.213
Intercept	8229.395	1	8229.395	151.284	0	0.341
Post hemoglobin	41.338	1	41.338	0.76	0.384	0.003
Group	4291.636	1	4291.636	78.895	0	0.213
Error	15883.91	292	54.397			
Total	254380	295				
Corrected total	20178.83	294				
Group	Post-fatigue estimated mean	Std. Error	95% confidence interval			
			Lower bound		Upper bound	
Experimental	24.345^a^	0.609	23.147		25.543	
Control	32.034^a^	0.611	30.832		33.236	

## Discussion

Hemodialysis helps to eliminate metabolic wastes only up to lethal concentrations. Therefore, interventions are essential to further enhance the clearance of metabolic wastes to reduce fatigue and improve the quality of life of affected patients. In this study, we observed that intradialytic aerobic exercise enhanced blood urea clearance among hemodialysis patients. The selected study participants of both groups were homogenous with respect to background characteristics. Our results indicated that the percentage of people with CKD undergoing hemodialysis increased with advancing age from 14.29% in those aged 21-30 years to 42.18% in those aged 51-60 years. Age-related muscle mass loss may eventually lead to a decline in glomerular filtration rate (GFR) in old age [11]. Additionally, studies have shown that the prevalence of CKD increased with age from 13.7% in those aged 30-40 years to 27.9% in those aged 70-80 years [12].

More than half of our study participants had a history of either diabetes mellitus or hypertension. In India, diabetes accounts for one-third of CKD cases (13%), followed by hypertension [13]. More than half of our study participants were male. For example, a meta-analysis of sex-stratified data showed that CKD development was quicker in men than in women [14]. This difference could also be due to a predominantly poorer lifestyle of smoking and drinking alcohol among men. The majority of our study participants were graduates, possibly indicating that CKD is a lifestyle disease influenced by improved educational and socioeconomic status. Our findings agree with those of Singh et al., who reported that CKD incidence is higher in those with a high school level of education, urban residents, overweight or obese adults, and moderate to high-income groups [15].

In our study, 38.10% (control group) and 35.81% (experimental group) had been receiving hemodialysis for more than three years. In India, the average life expectancy of a person receiving hemodialysis is less than three years, since 20 years [16]. These findings suggest that supportive interventions are essential to enhance urea and creatinine removal and increase life expectancy. Our study demonstrated an increased reduction in mean blood urea and fatigue score after dialysis in the experimental group compared to the control group. This result indicated the incremental effect of consistent compliance with intradialytic exercise in accelerating the excretion of urea during hemodialysis, which was lacking among the control group.

Our findings agreed with those of a similar study reporting significant differences in urea reduction rate (URR, p<0.001) between the control and treatment groups after eight weeks of treatment [17]. Our study observed no significant difference in mean post-test creatinine, potassium, or hemoglobin levels between the control group and experimental group (p>0.05), which agreed with the results of a pilot study by Paluchamy et al. [18]. However, their study found statistically significant differences in the mean values of blood urea, calcium, and phosphate between groups (p<0.05). Numerous studies have shown that intradialytic aerobic exercise improves dialysis parameters [19]. Muscle vasodilation caused by intradialytic exercise has been shown to enhance the elimination of solutes [20-22]. In one study, patients' levels of fatigue and serum phosphate, potassium, calcium, urea, and creatinine were found to be significantly reduced, with a modest increase in hemoglobin levels following eight weeks of an intradialytic range-of-motion exercise program; additionally, the experimental group's diastolic and systolic blood pressures were also found to be decreased [23].

In our study, no significant changes were noted in hemoglobin levels as a result of the exercise program, indicating the necessity to explore other approaches for substantial improvement. In another study, although an intradialytic exercise program gradually reduced fatigue, other physiological and biochemical indicators, such as potassium, urea, and creatinine levels, as well as systolic and diastolic blood pressure, did not change significantly [24]. The study concluded that a simplified physical exercise program may be considered a safe and effective clinical nursing modality in patients with end-stage renal disease receiving hemodialysis. The results of the study also indicated that although the decreased level of hemoglobin due to the disease process in hemodialysis patients significantly influenced fatigue levels, intradialytic exercise significantly reduced fatigue caused by urea accumulation. This effect prevents the sedentary lifestyle-related deterioration in physical function found among hemodialysis patients, which, at minimum, improves their prognosis, as physical activity results in improvement in physical function and well-being. The overall study findings indicate that intradialytic exercise can be incorporated as a safe and cost-effective nursing modality to improve the clearance of accumulated waste products and physical function among hemodialysis patients.

The outcome of this study was limited to certain biochemical parameters and fatigue. Due to financial constraints, repeated post-test assessment and analysis by repeated measure ANOVA were not possible. The study was restricted only to two tertiary care centers and eight weeks of intervention period due to feasibility and time constraints of the researcher. However, further studies are needed to replicate the study and generalize the findings.

## Conclusions

Our study found that an intradialytic exercise program is an ideal and safe modality for enhancing urea clearance during hemodialysis, as it requires no additional time or costs for the patient. Although aerobic fitness training has been shown to provide benefits for patients receiving hemodialysis, such programs have not gained widespread traction. Therefore, as a standard clinical practice, we suggest that an orientation and awareness program be established for nursing personnel working in dialysis units. Furthermore, randomized controlled trials, longer-term studies with larger sample sizes, investigations into a range of exercise regimens for hemodialysis patients, and studies investigating their impact on quality of life are required.
